# TRUS-MR Fusion Biopsy of the Prostate: Radiological and Histological Correlation

**DOI:** 10.5334/jbr-btr.1199

**Published:** 2016-11-24

**Authors:** Michel Lavaerts, Liesbeth De Wever, Els Vanhoutte, Frederik De Keyzer, Raymond Oyen

**Affiliations:** 1 Department of Radiology, UZ Leuven, campus Gasthuisberg, Belgium

**Keywords:** Fusion, Magnetic resonance imaging, Prostate biopsy, Prostate cancer, Ultrasound

## Abstract

**Objective:**

Targeted magnetic resonance/ultrasound fusion prostate biopsy has been shown to improve the detection of high-grade prostate cancer and to reduce sampling errors. Our objective is to assess MR-TRUS targeted fusion biopsy versus standard biopsy for the detection of clinically significant tumors.

**Materials and Methods:**

Patients were referred for abnormal digital rectal examination (DRE) or risen prostate-specific antigen (PSA). If an MRI-visible lesion was detected, they were included in the study. In total, 102 men underwent MRI followed by MR-TRUS fusion biopsy between November 2014 and January 2016. Tumor grading was done with the clinical relevance in mind; a cutoff was used at Gleason 7 or higher. Standard biopsy results were collected from clinical practice during 2005 at the same institution to provide baseline values.

**Results:**

A comparable rate of prostate cancer is found whether sampling is done at random (42.4%) or with the use of fusion biopsy (44.1%). However, these percentages are histologically different: fewer low-grade tumors are detected with MR-TRUS fusion biopsy (–19.1%), while more high-grade tumors are diagnosed (+26%). If there is an ultrasound-visible lesion in the prostate, the gain of combined MRI and fusion biopsy is less impressive.

**Conclusion:**

Fusion biopsy can provide more accurate information for optimal patient management, as it detects a higher percentage of high-grade prostate cancers than random sampling. Furthermore, nonrelevant tumors are less commonly detected using fusion biopsy.

## Introduction

Prostate cancer (CaP) is the most commonly diagnosed noncutaneous cancer and second-leading cause of death in men [1]. Many patients with organ-confined CaP undergo surgical therapy or radiotherapy, especially in high-grade CaP. However, low-grade cancers are more commonly stratified toward active surveillance [2,3]; therefore, the correct assessment of tumor grade is very important for patient management. Currently, the default assessment technique for the detection of CaP is based on lesion biopsy with histopathology. The most commonly used biopsy method is an at-random sampling of the entire organ. However, magnetic resonance imaging makes it possible to noninvasively assess CaP (with high sensitivity) before any invasive biopsy procedure is performed [3,4,5,6,7,8]. Because of this superiority of MRI imaging in lesion detection (compared to ultrasound), new biopsy methods have emerged that use this MR-obtained information during the procedure. In this setting, the possible approaches can be listed as follows [6]:

1. *At-random biopsy:* This is the current reference standard. Normally, 8 to 12 cores are sampled at random. This modality is standardized and is known for substantially undersampling the anterior and central part of the prostate, particularly in large prostates.
2. *Ultrasound targeted biopsy:* Whenever a lesion is apparent on ultrasound (US), one could selectively target it for biopsy. Most of the time, however, lesions are not visible on ultrasound, and the radiologist is forced back to option 1. In clinical practice, the radiologist usually combines at-random biopsies with targeted biopsy whenever possible.
3. *“Cognitive fusion”:* Even without dedicated fusion systems, prior MR imaging information can be used in the biopsy procedure. A trained practitioner is able to mentally “fuse” the MR information with the ultrasound images by identifying landmarks that are visible on both modalities. This is a method that is very operator- dependent.
4. *TRUS-MR targeted fusion biopsy:* This modality combines the superior diagnostic accuracy of MR imaging with the cost-effectiveness of transrectal US biopsy. The MRI images are superimposed on the TRUS images. The practitioner is able to take samples in predefined lesions.
5. *In-gantry “direct” MR-guided biopsy:* This is the only method that does not make use of ultrasound imaging. Because time on the MRI is still quite expensive, we think this time-consuming method is not yet ready for daily applications in CaP detection.

Comparison between literature reports is limited due to the variety of possible approaches and to the practitioner’s experience in these approaches (i.e., cognitive fusion). In this work, we will focus solely on the TRUS-MR fusion biopsy (approach 4) compared to the reference standard of at-random biopsies (approach 1).

The main purpose of this study is to examine whether a TRUS-MR fusion apparatus is beneficial to the outcome in CaP detection. More specifically, does fusion lead to detection of more clinically relevant and fewer low-grade tumors?

Secondary questions are whether there are means to reduce sampling errors. Or is there a lesion-volume threshold below which the accuracy of the fusion apparatus is insufficient?

## Materials and Methods

### Population

Patients in this study were men with elevated PSA or an abnormal DRE with at least one lesion in the prostate on MRI. They were prospectively enrolled at UZ Leuven, Belgium, in a clinical trial with ethical commission approval (registration number: mp07480). Enrollment occurred between November 2014 and January 2016 with written informed consent (15-month period). More than half (51,5%) of all included patients had had prior negative at-random biopsies. One must note that this is just a mere finding; this had nothing to do with our inclusion criteria. For a lot of patients, their initial investigation was in fact the MRI included in this study. All data was retrospectively analyzed in the framework of this master paper.

### Inclusion criteria

Every patient with lesions on MRI was included. Those without lesions received at-random biopsies and were excluded from this study (their outcome is outside the scope of this fusion-oriented study). Individual patient inclusion was decided on a per-case basis by the attending radiologist, without fixed lesion volumetric threshold in the beginning. Approximately halfway through the study, we had the impression that the very small (< 0.2 cc) regions of interest (ROI) nearly always seemed to be histologically insignificant. The study inclusion criteria—for a lesion—was therefore altered to only include larger (> 0.2 cc) lesions on MRI, most of which are still really small. Half of the ROIs measured less than 0.4 cc, of which most were sampled in the beginning of the study. Only 25 percent of the ROIs measured more than 1.9 cc. Exclusion criteria were prior prostate surgery or cancer therapy and contra-indication to MRI in general.

### Methods

#### Imaging Protocol

All patients underwent MRI on a Siemens Magnetom Aera 1.5 Tesla system for a total scan time of around 15 minutes. We used an 18 phased**-**array body coil. The T2 sequences (Table 1) are used to localize and view the prostate anatomy as well as stage possible tumoral lesions. Because inflammation can mimic CaP, diffusion-weighted images (DWI) were added to identify possible malignant ROIs [9]. DWI quantifies free water movement, known as “Brownian” motion. In tumoral lesions, the increased cellularity reduces the water mobility, leading to “restricted diffusion,” which is characterized by a low apparent diffusion coefficient (ADC) [10,11]. The degree of diffusion restriction is known to correlate with the Gleason score, probably reflecting that same cellular-density aspect [12,13]. One T1 sequence was added to rule out previous biopsy damage (and as such rule out false-positive results). Lesions on MRI were delineated by a radiologist, assigned a grade of CaP suspicion, and imported into the fusion system for biopsy using the ProFuse software.

**Table 1:** Imaging protocol.

Parallel imaging technique: Grappa, Factor 2								

T1/T2	Orientation	Acquisition time	Pixel size in mm.	# slices	Field of View (mm)	Slice thickness (mm)	Repetition time (ms)	Echo Time (ms)

T2	Sagittal	1:49	0.6*0.6	26	260 * 260	3.5	7700.0	133.0
DW	Transversal	5:47	2.7*2.7	42	350 * 285	4.0	9900	67.0
T2	Transversal	5:05	0.6*0.6	56	260 * 236	3.0	11250.0	124.0
T2	Coronal	2:50	0.6*0.6	40	260 * 260	3.5	14010	124.0
T1: fat suppression	Transversal	0:18	1.0*1.0	52	320 * 260	3.0	4.26	2.09

#### Biopsy Protocol

**Figure 1:**
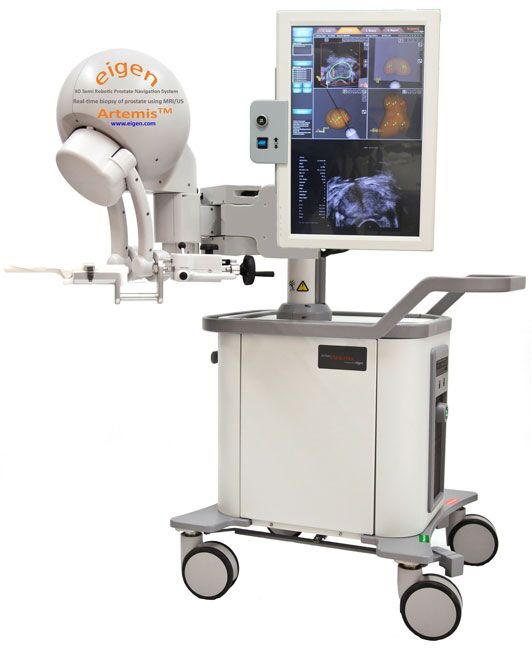
Artemis Eigen Fusion system [14].

The Artemis device (Eigen, Grass Valley, CA) is a 3D ultrasound-guided prostate biopsy system that provides tracking of MR-identified ROIs within the prostate on MR-imagery fused with real-time ultrasound [15] (Figure 1). The radiologist who delineated the ROIs on the MRI images was the same physician that performed the biopsy.

**Figure 2:**
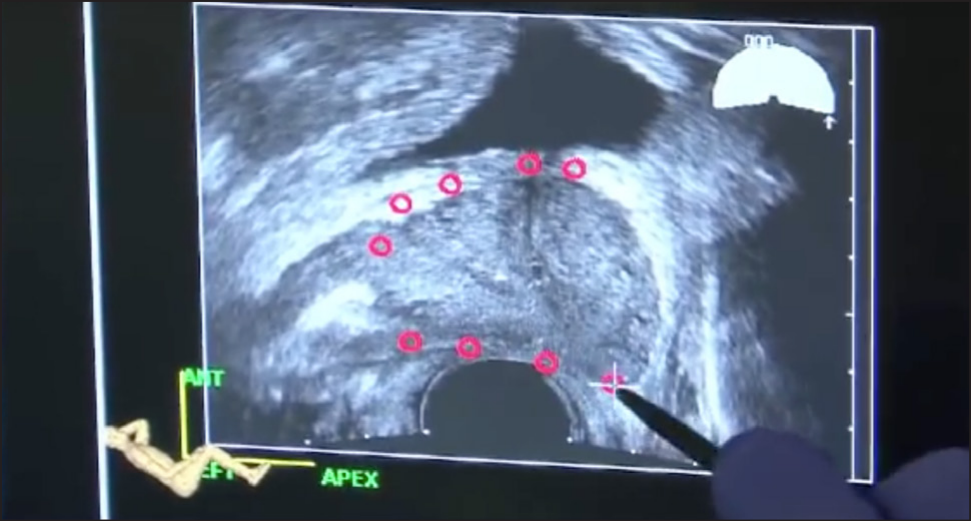
Outlining of the prostate on the Artemis system.

A prophylactic antibiotic was given, and the procedure was conducted with the patient in left lateral decubitus. The prostate was anesthetized with a peri-prostatic block. The radiologist then made a complete axial transrectal ultrasound volume sweep of the prostate and outlined the outer contours on the fusion system (Figure 2). The process of acquiring the US sweep, delineating the prostate margin, and calculating the software fusion takes around 5 minutes, while the biopsy procedure itself, on average, took 15 minutes. Sampling was done with a conventional spring-loaded gun inserted in the fusion apparatus. A recalibration of the fusion images was done each time the patient had moved.

The procedures were executed by one of three radiologists from the same hospital, with 2 (EVH), 10 (LDW), and 30 years (RO) of experience in genitourinary radiology.

**Figure 3:**
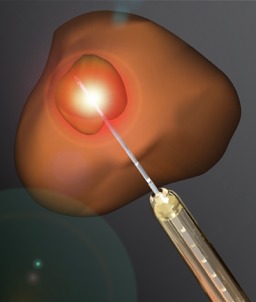
Precise sampling of a ROI [25].

In this study, only biopsies were taken of the ROIs which we had previously outlined on the MRI images (Figure 3). There was (mostly) no concomitant at-random biopsy performed.

#### Comparison

In 2005, there had been a study at our institution on at-random sampling of the prostate. We wanted to compare our current results of fusion biopsy with the at-random results of that study. Although inclusion criteria are very similar in both studies (risen PSA and/or abnormal DRE), it is important to note that, in 2005, patients were not screened yet on MRI, so no information was available on whether any lesions were visible on MRI. This could lead to a slightly different inclusion bias.

### Statistical analysis

**Figure 4:**
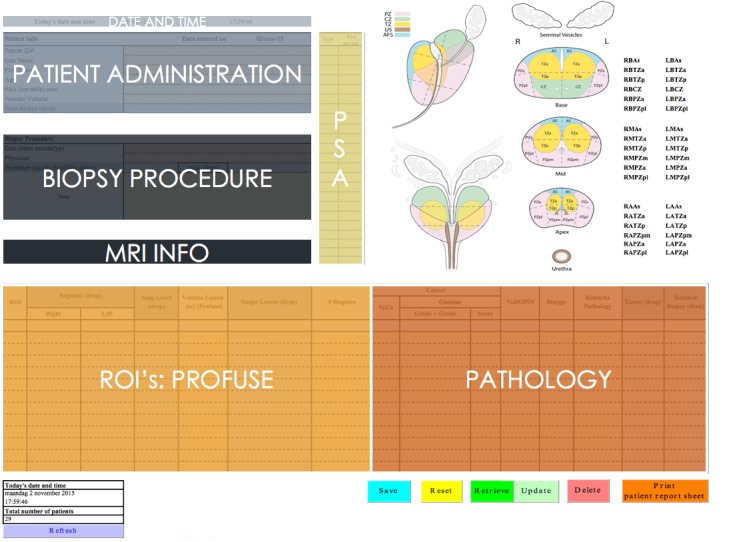
Study database set-up.

Each lesion was identified as a separate ROI with a specific histological outcome (Figure 4: database). In patients where more than one tumor focus was found, the highest Gleason score was reported.

Analysis was done by a statistician of the department of bio-statistics at UZ Leuven. The Spearman correlation was calculated as a measure of association between two continuous or ordinal variables. The Mann-Whitney *U* test was used for testing the difference between two groups on continuous or ordinal variables. Further logistic regression models were used to analyze binary outcomes as well as proportional odds models for ordinal outcomes. The at-random results of 2005 were compared with the current fusion results using the chi-square test. All tests are two-sided, and a 5 percent significance level is assumed for all tests. Analysis was performed using the SAS software (version 9.4 of the SAS System for Windows).

#### Two Different Risk Stratifications Were Used

The first risk stratification was to compare our results with our own at-random database from 2005. Here we’ve chosen to look solely at the Gleason scores. This risk stratification is the most relevant for patient management and outcome. Clinically, the Gleason scores of 7 or higher are of real significance [2,3,16]. We therefore subdivided our patients in clinically “high-grade tumor” (above Gleason score of 6) or “low-grade tumor” (below or equal to Gleason score of 6) groups.

The second risk stratification used was to compare our results with the well-known JAMA study by Siddiqui et al. [17] in which “low-risk JAMA” on biopsy was defined as Gleason score 6 or low-volume Gleason score 3+4 (i.e., < 50% of any core containing cancer); “intermediate-risk JAMA” was defined as Gleason score 3+4 with 50 percent or more of any core positive for cancer; and “high-risk JAMA tumors” were defined as Gleason score 4+3 or greater.

## Results

### Per-patient Results

Patients included in the study were 102 men aged 52–80 (66.2 ± 6.5 years), with a mean PSA of 9.5 μg/l ± 6.2 μg/l) (Table 2). The median time between MRI and biopsy was 23 days (range: 0–210 days).

**Table 2:** Patient demographics.

Variable	Statistic	All

**Age**	N	102
	Mean	66.2
	Std	6.56
	Median	67.5
	IQR	(61.0; 71.0)
	Range	(52.0; 80.0)
**PSA**	N	102
	Mean	9.5
	Std	6.23
	Median	7.1
	IQR	(5.8; 11.8)
	Range	(1.1; 38.0)
**Prostate Volume**	N	102
	Mean	5837.3
	Std	58305.12
	Median	60.5
	IQR	(40.1; 80.7)
	Range	(4.9; 588917)
**Prior Biopsy**		
No	n/N (%)	48/99 (48.48%)
Yes	n/N (%)	51/99 (51.52%)
**Tumor risk group (according to JAMA article)**		
No tumor	n/N (%)	57/101 (56.44%)
Low risk	n/N (%)	21/101 (20.79%)
Intermediate risk	n/N (%)	9/101 (8.91%)
High risk	n/N (%)	14/101 (13.86%)
**Highest Gleason score**		
No tumor	n/N (%)	57/102 (55.88%)
3	n/N (%)	1/102 (0.98%)
6	n/N (%)	16/102 (15.69%)
7	n/N (%)	20/102 (19.61%)
8	n/N (%)	7/102 (6.86%)
9	n/N (%)	1/102 (0.98%)

Regarding the PSA relation to patient age, Spearman correlation showed that PSA increases significantly with patient age (*p* = 0.006). Whether or not carcinoma was found showed no significant correlation with the absolute PSA value (*p*-value = 0.34) nor with patient age (*p*-value = 0.43).

A slight majority (51.5%) of patients had had prior at-random biopsies that were all histologically negative. If a patient underwent more than one fusion-guided biopsy, only the first session was evaluated in our analysis.

**Figure 5:**
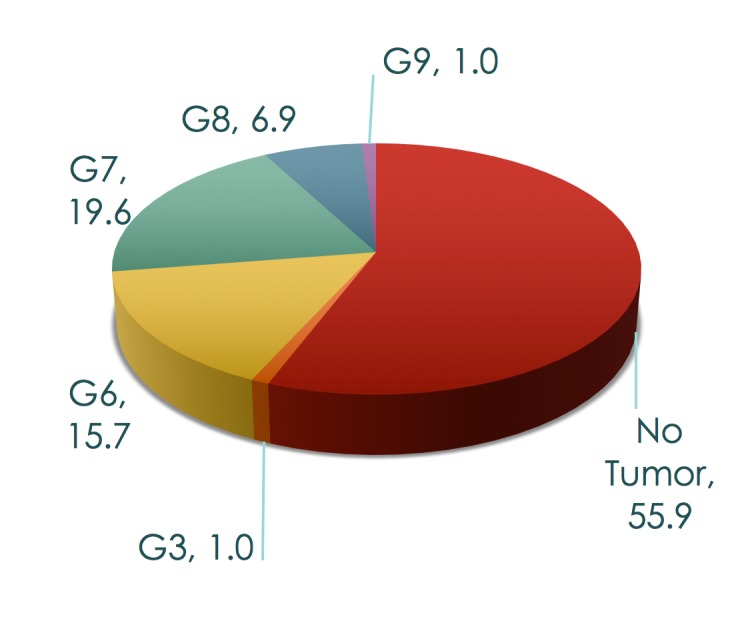
Fusion cancer detection rate per Gleason score (in percentages).

In our study, 55.9 percent of patients had no focus of tumor, while 44.1 percent did test positive for CaP (Figure 5).

### Per-lesion Results

**Figure 6:**
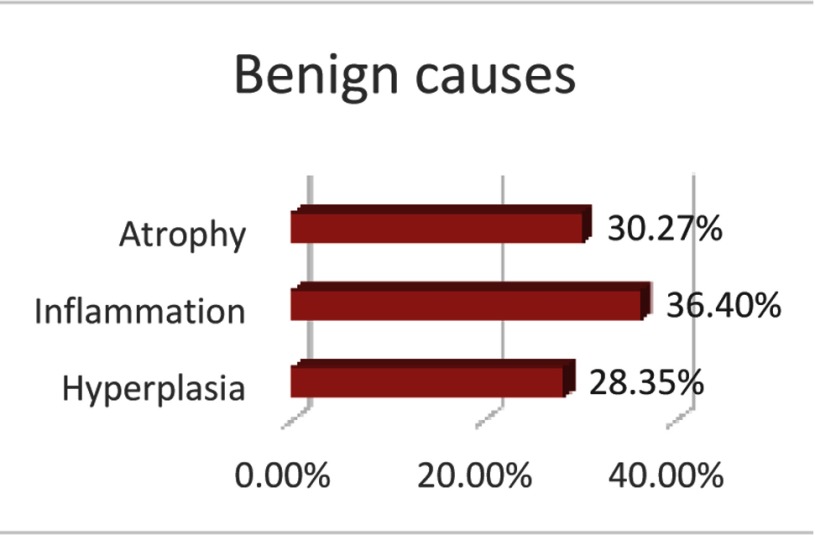
Benign causes of diffusion restriction.

In total, 341 regions of interest (ROI) were outlined and sampled in 102 patients, and, on average, 2.4 biopsies (±1.45) were taken per ROI. Of the 341 ROIs that were biopsied, 80 were found histologically positive for adenocarcinoma, and 261 samples tested negative on carcinoma, of which 70.9 percent showed one or more benign cause. When benign cause was identified, it was mostly attributed to atrophy, inflammation, hyperplasia, or a combination of these (Figure 6).

### Higher Detection Rate Of High-Grade Tumors, Fewer Detection Of Low-Grade Tumors

**Figure 7:**
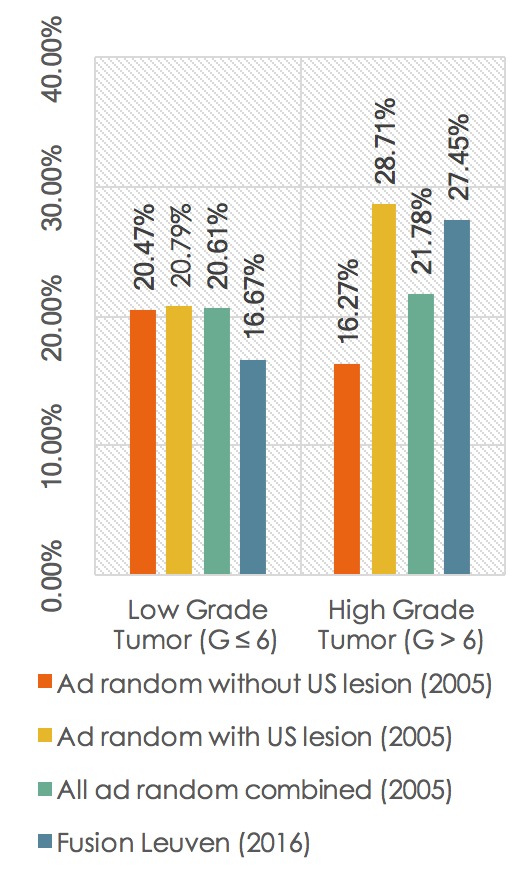
Fusion biopsy versus at random sampling.

In 2005, we biopsied 303 men at random that had ultrasound-visible lesions in the prostate and 381 men that had no ultrasound visible lesion. Only the truly at-random biopsy results are included in our comparison. To be clear, there was no US-targeted biopsy result included, not even when lesions were visible on ultrasound (Figure 7 + Table 5).

#### Fusion Biopsy versus “At-random Biopsy When No Lesion Is Visible on Ultrasound”

We have found 18.6 percent less low-grade tumors with fusion biopsy and 68.7 percent more high-grade tumors (*p*-value of 0.01) compared to the at-random biopsies from 2005 where no lesion was seen on ultrasound.

#### Fusion Biopsy versus “At-random Biopsy When a Lesion Is Visible on Ultrasound”

With fusion biopsy, we detect 19.8 percent fewer low-grade tumors and 4 percent fewer high-grade tumors compared to the at-random biopsies of 2005 where ultrasound visible lesions were seen. In that group, the fusion biopsy results are inferior compared to the at-random sampling results. We do know, however, that lesions that are visible on ultrasound have a higher chance of being malignant [18].

When all at-random cases of 2005 are combined, fusion biopsy finds 19.1 percent less low-grade tumors and 26.0 percent more high-grade tumors compared to the at-random technique.

**Figure 8:**
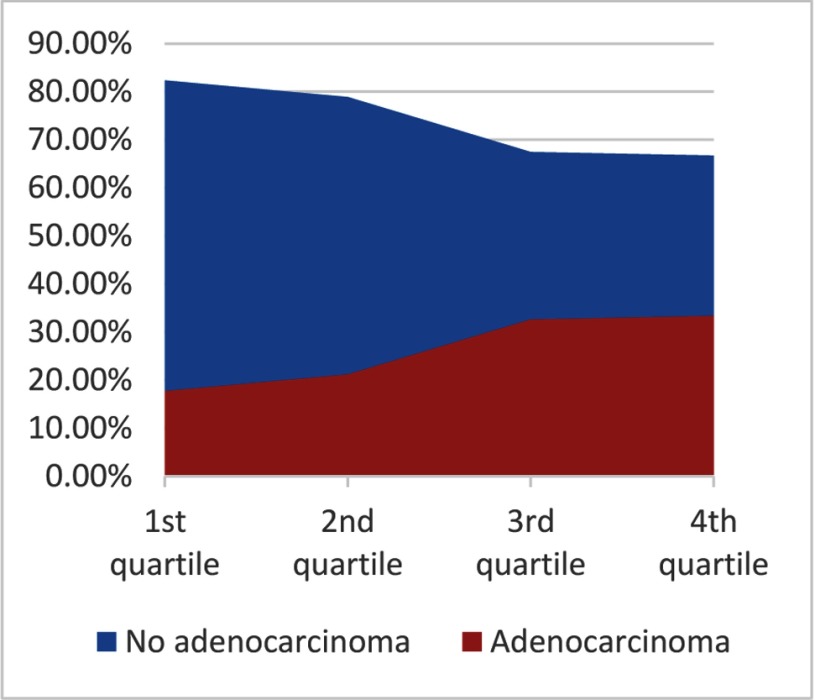
Frequency of carcinoma detection.

One can notice an increase in detection somewhere halfway in our study (*p*-value: 0.0643) (Figure 8), around the time the patient selection criteria were altered (we stopped sampling lesions < 0.2 cc).

Biopsy procedures were performed by three different physicians that each accounted for 9 percent (RO), 39 percent (LDW), and 52 percent (EVH) of the interventions. No significant differences were observed in terms of outcome between practitioners (*p*-value: 0.1374). One must note that the relatively small population makes it difficult to definitely rule out inter-observer variance.

No statistical significant correlation was found between ROI volume (nor ROI volume %) and the histological outcome (*p*-value 0.0967 and 0.5285, respectively).

## Discussion

In this study, targeted biopsy significantly increased the detection of clinically significant tumors (> Gleason score 6), and fewer low-grade tumors were detected.

A higher detection rate of high-grade tumors will lead to more clinically relevant information for the urologist’s daily practice. This implies better sensitivity and specificity of the detection modality and is therefore beneficial for patient management. If high-grade tumors are detected earlier, the gain in time will be translated into an earlier start of therapy. Patients with a Gleason score above 6 will more likely be accurately detected and, therefore, less likely be mistakenly stratified toward active surveillance because of suboptimal samples. Because of higher specificity, when biopsy returns negative, there will be less need for resampling or early follow-up, and, in the end, fewer MRI scans will be necessary.

Low-grade tumors (≤ Gleason score 6) are most often not treated immediately because it has been shown that CaP mortality (at 7 years) is not significantly different between immediate radical prostatectomy and active surveillance [2,3]. Therefore, the lower detection rate of low-grade tumors with fusion biopsy is not necessarily a negative finding and might even be called positive as it avoids unnecessary treatment and limits patient anxiety. In patients with multiple lesions, this approach also allows to minimize the required number of biopsy cores by only focusing on the lesions with the highest (expected) Gleason score.

Adding standard biopsy to targeted biopsy, according to Siddiqui et al. [17] leads to a 22 percent higher detection rate of cancer. However, 83 percent of these are low risk, while only 5 percent are high risk. This equates to the need to biopsy 200 men to diagnose 1 additional high-risk cancer. Differently stated, to obtain 1 additional high-risk tumor, 17 additional cases of low-risk cancer would be diagnosed. The incorporation of standard biopsy in addition to targeted biopsy led to no change in Gleason score risk stratification in 85 percent of patients in their study. Therefore, one can state that no concomitant at random biopsy needs to be performed.

The detection rate went up during our study, even though experience with the biopsy system is one attributing factor to that effect; the main cause is the change of inclusion criteria midstudy (Figure 8). Most small lesions came back negative, which gave us the idea that they were too small to sample precisely. It made us question the accuracy of the approach. Because of the size of our study, we can only conclude that there is an increased detection rate over time with a significant leap upward when the inclusion criteria became more stringent. However, the current data is not sufficient to estimate the optimal volume threshold for successful biopsy.

A study from 2011 tracked biopsy accuracy in 11 consecutive men by removing, detaching, and cleaning the ultrasound probe after biopsy was done and then reinstalling the complete setup while the patient remained still. They then re-biopsied three randomly selected sites in each patient, and the distance between original and re-biopsy sites was determined. The mean error for all 32 biopsies was 1.2 ± 1.1 mm (range 0.2–5.1) and was independent of prostate volume or biopsy location [19]. This might seem very accurate, but it needs some extra explanation.

These numbers represent solely the accuracy of repeating biopsies in the same lesions. This error rate stands side by side with the already existing error margin made by the fusion process itself (which is multifactorial: time between MRI and biopsy procedure, operator delineating experience and proficiency, patient movement). Therefore, we advise to not sample the millimetric lesions, as accuracy cannot be guaranteed (yet).

If a lesion is visible on ultrasound, the differences between at-random biopsies and fusion biopsies is negligible. With fusion, we detected slightly fewer low-grade tumors and fewer high-grade tumors compared to at-random sampling ultrasound-visible lesion prostates. It seems, therefore, that a fusion biopsy should no longer be performed if an ultrasound-visible lesion is detected and the practitioner is certain that it is the same lesion as seen on MRI. In that case, solely ultrasound targeted and at-random biopsy will suffice. If the practitioner is not sure whether it is the same lesion as seen on MRI, it seems prudent to complete the fusion biopsy as well as the visually targeted and at-random biopsy to obtain optimal results.

No significant differences were seen in our study between the observed frequencies of tumors per segment; however, a tendency exists that more tumors originate from locations outside of the transition zone (Table 3). This same trend was seen when compared to the study of 2005 (Table 4). Both studies are comparable in terms of where tumors originate from.

**Table 3:** 2015 Fusion biopsy: observed frequencies of tumors per segment (transition zone TZ versus non-transition zone nTZ).

Right-TZ	Right-nTZ	Left-TZ	Left-nTZ

22.37% (17/76)	27.63% (21/76)	19.74% (15/76)	30.26% (23/76)

**Table 4:** 2005 At random biopsy: observed frequencies of tumor detection per segment.

	Transition zone	Non-transition zone

**US-visible lesion**	41.44%	58.55%
**No US-visible lesion**	41.84%	58.15%

**Table 5:** Comparison with the at random results from 2005.

Gleason score	Modality	Result 2005: at random sampling	Result 2015: Fusion biopsies	P-value

All	without US lesion	140/381 (36.75)	45/102 (44.12)	0.1737
All	with US lesion	150/303 (49.50)		0.3463
All	all at random (sum)	290/684 (42.40)		0.7432
G<=6	without US lesion	78/381 (20.47)	17/102 (16.67)	0.3904
G<=6	with US lesion	63/303 (20.79)		0.3654
G<=6	all at random (sum)	141/684 (20.61)		0.3534
**G>6**	**without US lesion**	**62/381 (16.27)**	**28/102 (27.45)**	**0.0100**
G>6	with US lesion	87/303 (28.71)		0.8069
G>6	all at random (sum)	149/684 (21.78)		0.2012
P-values from chi-square test				

## Reducing sampling errors

Adequate loco-regional anesthesia is very important to reduce sampling errors. Low patient pain will lead to less movement and a higher biopsy accuracy, as each (significant) patient movement forces the radiologist to perform a recalibration step in the fusion system. Each recalibration step increases the total procedure time, which decreases anesthesia efficacy and increases patient discomfort and mobility. Because of this, a total recalibration after each movement is practically impossible, and patient movement will therefore lead to inaccuracy.

## Current literature

The idea that more high-grade and fewer low-grade CaP is detected by fusion biopsy is made fact by a study published in *Oncotarget* in August 2015. This study is a meta-analysis of 16 paired cohort studies by Wu et al. [20]. They compared all known targeted-fusion biopsy articles available to date (before August 2015). There were a total of 3,105 patients studied. It showed that MR-TRUS fusion biopsy detects more clinically significant cancers than systematic biopsy with great statistical significance (RR = 1.19; *p*-value < 0.05). We had the same conclusions with comparable outcome in terms of percentages.

**Figure 9:**
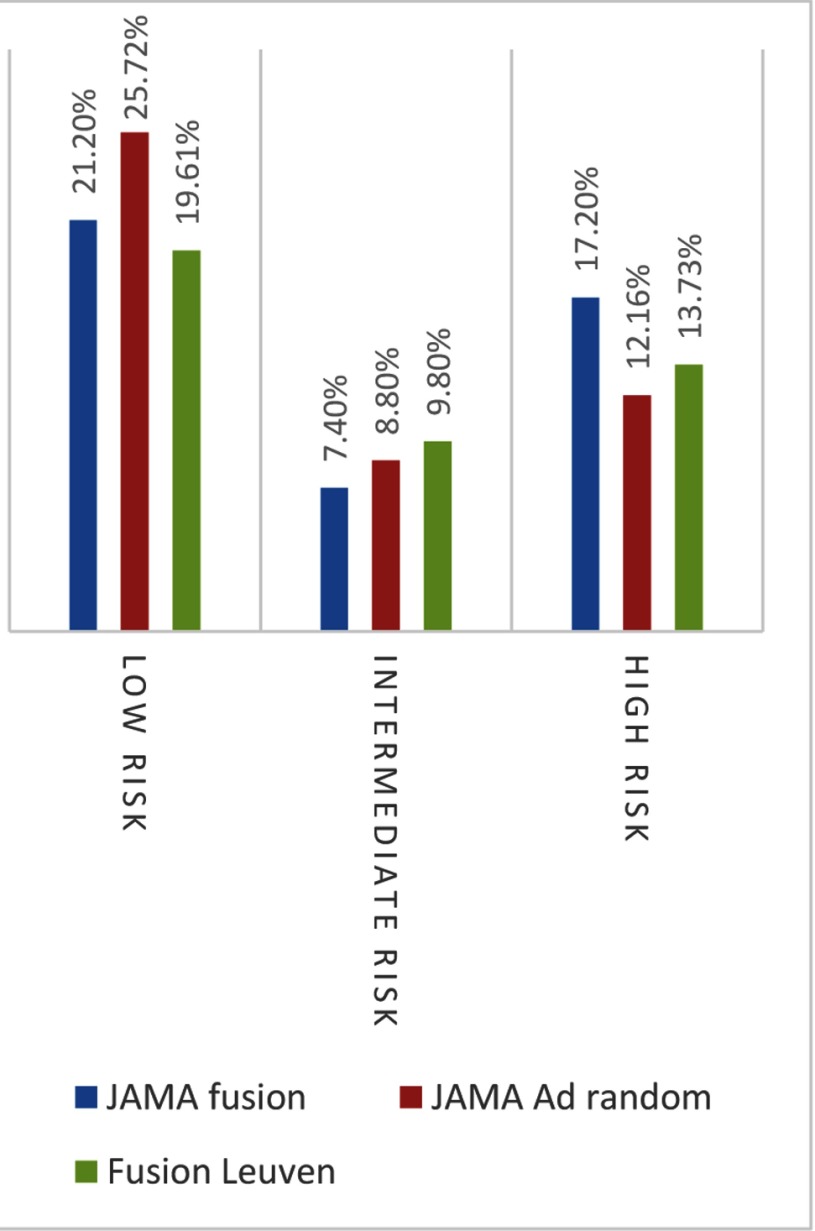
Our fusion results versus their at random/fusion results.

One of the main contributors to this meta-analysis was the study performed in 1,003 patients by Siddiqui et al. When we compare our data using the same risk stratification (see chapter “Statistical Analysis”) as they had used in their study, we could categorize 19.6 percent in the low-risk JAMA group, 9.8 percent in the intermediate-risk JAMA group, and 13.7 percent in the high-risk JAMA group. Below is a graphical comparison of these results (Figure 9). To compare with the JAMA study, one patient was excluded from the calculations because of the unknown carcinoma percentage in a lesion. The limitation of this comparison is that we were not able to include our own at-random results in this graph because, in 2005, we had not included lesion size in our database, so the percentages in the next paragraph will be our results in correlation to their at-random cases.

They found 17.5 percent fewer low-risk carcinomas, while we found 23.7 percent fewer low-risk CaP. And 41.5 percent more high-risk tumors were diagnosed in the JAMA article, while we had a mere 12.9 percent increase in high-risk tumor detection.

The reason these numbers vary is presumably multifactorial, related to differences in inclusion criteria and the number of included patients. While our results are a bit different than theirs, especially due to the lower prevalence of higher Gleason scores in our study, the same trend was found in both studies: fewer low-risk tumors were detected, and more high-risk tumors were diagnosed.

## Limitations

The main limitation in our comparison with the at-random results of 2005 is that, back then, there was no MRI screening indication for the prostate, so even the people without lesions were biopsied. That means that the results of this study represent a combined effect: the efficiency of fusion biopsy as well as the lesion detection of MRI. On average, the JAMA study showed that around 15 percent of people that underwent MRI showed no lesions [17]. We were not able to verify this for our own population. However, we can expect comparable numbers at our institution (because of the size of their study). Not performing an at-random biopsy in the cases where MRI did not show a lesion is justified by the fact that the negative predictive value of a negative MRI is 98 percent for a Gleason score 7 lesion or higher, the same cutoff we used in our study [21]. So although the MR-inclusion criteria causes a bit of a bias, the same trend toward a higher detection of high-grade tumors remains.

Prostate MRI was performed without endorectal coil (ERC). The current PIRADS version 2 guidelines state that contemporary 1.5 T scanners that employ a relatively high number of external phased array coil elements and RF channels (16 or more) “may be capable of achieving adequate signal-to-noise ratio in many patients without an ERC.” Because our equipment complied to these criteria as well as patient comfort, this study was performed without ERC [22].

One other limitation might be that we did not perform multiparametric MRI (MP-MRI), which implies the use of at least two functional imaging techniques. Mostly DWI as well as dynamic contrast-enhanced sequences (DCE) are then used. We only performed T2-weighted (T2W) imaging in combination with DWI. However, the detection rate is significantly higher for T2W + DWI than that for T2W + DCE or the three sequences combined. Especially in the transition zone, the MP-MRI model fails to improve the detection rate compared to T2W and DWI sequences alone [23]. Therefore, conducting a complete MP-MRI protocol was, in our understanding, of no additional value in this study.

Even though all previously listed findings translate into great clinical benefits, it is important to recognize that this study is preliminary toward prostate cancer–specific mortality.

The cost and net benefit of giving every patient an MRI as well as fusion biopsy needs to be investigated in further studies. There is however a study that investigated in-gantry MR-biopsy that concluded that when the benefits of MRI were considered, the expected costs per patient, in the end, were virtually the same [24].

## Conclusion

Fusion biopsy significantly increased the detection rate of clinically significant tumors and decreased the detection rate of low-grade tumors. A higher detection rate of high-grade tumors will lead to more clinically relevant information and is therefore beneficial for patient management. If high-grade tumors are detected earlier, the gain in time will be translated into earlier therapy. Because of higher specificity, there will be less need for resampling and less need for early follow-up.

The lower detection rate of low-grade tumors might be called positive as it avoids unnecessary treatment and limits patient anxiety. In patients with multiple lesions, this approach also allows us to minimize the required number of biopsy cores by only focusing toward the lesions with the highest (expected) Gleason score.
